# The Relationship between Mold Exposure and Allergic Response in Post-Katrina New Orleans

**DOI:** 10.1155/2010/510380

**Published:** 2010-06-16

**Authors:** Felicia A. Rabito, Sara Perry, W. Edward Davis, C. Lillian Yau, Estelle Levetin

**Affiliations:** ^1^Department of Epidemiology, Tulane University School of Public Health and Tropical Medicine, 1440 Canal Street SL-18, New Orleans, LA 70112, USA; ^2^Allergy and Immunology, Ochsner Health System, 1514 Jefferson Highway, Jefferson, LA 70121, USA; ^3^Department of Biostatistics, Tulane University School of Public Health and Tropical Medicine, 1440 Canal Street, New Orleans, LA 70112, USA; ^4^Faculty of Biological Science, The University of Tulsa, 600 S. College, Tulsa, OK 74104, USA

## Abstract

*Objectives*. The study's objective was to examine the relation between mold/dampness exposure and mold sensitization among residents of Greater New Orleans following Hurricane Katrina. *Methods*. Patients were recruited from the Allergy Clinic of a major medical facility. Any patient receiving a skin prick test for one of 24 molds between December 1, 2005 and December 31, 2008 was eligible for the study. Exposure was assessed using standardized questionnaires. Positive mold reactivity was defined as a wheal diameter >3 mm to any mold genera. *Results*. Approximately 57% of participants tested positive to any indoor allergen, 10% to any mold. Over half of respondents had significant home damage, 34% reported dampness/mold in their home, half engaged in renovation, and one-third lived in a home undergoing renovation. Despite extensive exposure, and multiple measures of exposure, we found no relationship between mold/dampness exposure and sensitivity to mold allergens. *Conclusions*. These results along with results of earlier research indicate no excess risk of adverse respiratory effects for residents living in New Orleans after the devastation of Hurricane Katrina.

## 1. Introduction

Residing in damp or water damaged homes is closely associated with observations of mold, mildew, and other microbial growth and has been associated with respiratory illnesses [1–4]. The Institute of Medicine concluded that there is a causal link between indoor dampness and upper respiratory tract symptoms, cough, wheeze, and asthma symptoms in sensitized people, and hypersensitivity pneumonitis in susceptible people [5]. An estimated 21% of current asthma is attributable to dampness/mold in homes [6]. Many molds produce IgE-inducing allergens and studies have shown higher prevalence of mold sensitization among people living in damp homes and homes with elevated concentrations of molds [7–9]. 

Molds and other fungi may adversely affect human health through allergy, infection, and toxicity. Approximately 40% of the population is atopic and express high levels of allergic antibodies to inhalant allergens [10]. In atopic individuals, inhalation of allergens can trigger an immune response characterized by eosinophilic inflammation, constriction of the airways, and increased levels of IgE antibodies [11, 12]. An estimated 50–75% of asthma cases are attributed to atopy and 10% of the population has allergic antibodies to fungal antigens [10]. Despite evidence that indoor dampness/moldiness is associated with respiratory conditions, little is known about the contribution of indoor mold levels to allergic sensitization rates [13, 14]. 

Hurricane Katrina struck the New Orleans area in August 2005. Widespread damage resulted in substantial environmental degradation. In New Orleans, 80% of the city and 120,000 homes flooded [15, 16]. Exposure assessments after the storm detected culturable levels of indoor mold at concentrations between 22,000 to 515,000 CFU/m^3^, well above the threshold associated with adverse health effects [15–17]. Mycotoxins have been detected in home dust samples two years after the storm [18]. These data coupled with qualitative reports of increased respiratory and allergic illness in the general population led to concern about adverse effects of exposure to mold both in the short term and in the long term [19]. Despite evidence of increased ambient exposure and qualitative reports of increased allergic response, to our knowledge there has been no study assessing the relationship between the two. Additionally, the long term effect of living in an environment subsequent to massive flooding remains undescribed. 

The purpose of this study was to examine the relation between reported exposure to mold/dampness and mold-specific allergic sensitization among residents of the Greater New Orleans area following Hurricane Katrina. The hypothesis being tested is that persons with higher levels of mold or dampness exposure are more likely to be mold sensitized than those with lower levels of exposure and that due to the extent of exposure the prevalence of mold reactivity in the Greater New Orleans area would exceed the national average.

## 2. Materials and Methods

We conducted a 3-year study of patients presenting to the Allergy, Asthma and Immunology clinic of the Ochsner Health System, a major medical facility in the New Orleans area. The source population was patients receiving a skin prick test between December 1, 2005 and December 31, 2008. Any patient skin prick tested for mold reactivity was eligible for the study. Skin prick testing on patients presenting to the Clinic is performed to evaluate potential allergic disease in patients complaining of respiratory difficulty, rhinitis, conjunctivitis, cough, wheezing, or dypsnea. Patients presenting to the clinic between January 1, 2008 and December 31, 2008 were prospectively recruited by clinic nurses. Patients who underwent skin prick testing between December 2005 and December 31, 2007 were retrospectively recruited via mail using contact information contained in clinic records. All eligible participants received up to three recruitment letters and a recruitment postcard encouraging participation in the study. Those agreeing to participate completed an informed consent and an exposure assessment survey. 

### 2.1. Exposure Assessment

Exposure to mold or dampness in both the home and workplace was assessed via survey questionnaire. Questions were adapted from validated survey tools and measured exposure through multiple domains [20–22]. Questions relevant to exposure and pertinent to Hurricane Katrina (e.g., number of residences and extent of damage) were also included on the exposure assessment questionnaire.

Using a standard mold/dampness self-reported exposure format, participants were asked the following five exposure questions; since Hurricane Katrina (1) have you observed water damage, water leaks, damp stains, standing water, or condensation in the home, (2) have you noticed a musty odor in your home, (3) have you observed mold or mildew in your home, (4) have you observed mold or mildew inside your workplace, (5) have you worked in an occupation where you were regularly exposed to mold. To assess length of exposure, participants were asked to estimate the number of months associated with each of the five exposures listed above. Questions regarding the extent of hurricane-related damage, renovation activity, and use of personal protective equipment were also included. Extent of home repair was measured on an ordinal scale; 0 = no need for repair, 1 = cosmetic repairs only (painting or cleaning only), 2 = surface material repairs (painting or cleaning plus new carpet, sheetrock, etc.), and 3 = structural repairs (part or all of the house gutted). Finally, questions were asked about previous allergy testing, prevalence of asthma, and the nature of previous visits to the Ochsner Allergy clinic. 

Exposure was assessed three ways. First, an ordinal scale was constructed similar to the one used by Engvall et al. and based on the number of “yes” responses to the five exposure questions [23]. Exposure was also categorized as either minimally exposed (yes to one exposure question), moderately exposed (yes to 2 or 3 questions), or highly exposed (yes to 4 or 5). Finally, the total months of exposure from all five questions was used as a continuous variable for exposure. We also looked at renovation activities as a potential source of mold exposure. The mean number of months spent renovating a home or living in a home while it was being renovated was assessed along with the use of personal protective equipment.

### 2.2. Skin Testing

Mold allergens used for testing included *Acremonium*, *Alternaria alternata*, *Aspergillus fumigatus*, *Botrytis cinerea*, *Candida albicans*, *Chaetomium globosum*, *Cladosporium cladosporioides*, *Curvularia spp.*, *Epicoccum nigrum*, *Fusarium ssp.*, *Gliocladium*, *Helminosporium*, *Mucor spp.*, *Neurospora spp.*, *Nigrospora spp.*, *Penicillium Mixed*, *Phoma herbarum*, *Pullularia pullulans*, *Rhizopus spp.*, *Rhodotorula rubra*, *Smuts Mixed*, *Stemphyllium spp.*, *Trichoderma*, and *Trichophyton mentagrophytes*. All extracts were glycerinated and supplied by ALK-Abello, Inc. (Round Rock, TX, USA) in a 1 : 20 weight/volume concentration except *Gliocladium* (1 : 20 weight/volume) and *Trichoderma *(1 : 40 weight/volume) were supplied by Greer Labs (Lenoir, NC, USA). Histamine (10 mg/ml) and saline were used as positive and negative controls. Drops of the allergens were applied to the back and a single lancet (AccuSet, Alk-Abello, Inc., Round Rock, TX) was passed through each drop to prick the skin. Tests were read 15 minutes later using the following scale: negative, 1+ (erythema only), 2+ erythema with wheal ≤3 mm, 3+ erythema with wheal >3 mm, and 4+ Erythema with pseudopods. Cases of positive mold reactivity were defined as a wheal diameter >3 mm to any mold genera.

### 2.3. Statistical Analysis

The analysis involved descriptive statistics, unadjusted estimates and tests of, including chi-square and *t*-tests, crude odds ratios and 95% confidence intervals, and modeling techniques to adjust for relevant covariates. Logistic regression methods were used to determine the relationship between the mold positivity and dampness/mold exposure, controlling for potential covariates.

## 3. Results

A total of 529 patients presenting to the Allergy and Immunology clinic between December 1, 2005 and December 31, 2008 were tested for mold reactivity and completed an exposure assessment questionnaire. Demographic, residential, and clinical characteristics of the study population are described in Table 1. The majority of participants were white (78.7%) and female (67.1%). The median age was 44 years and participants ranged in age from 1 to 93 years. Forty-seven percent of respondents lived in at least two residences since Hurricane Katrina. Over half (53.3%) of respondent homes required at least surface material repairs. Over half (56.7%) of all patients tested positive to at least one allergen. Thirty-four percent tested positive to either Der p1 or Der f1; 27.6% to any tree pollen; 22.5% to any weed pollen; 26.6% to any grass pollen. Doctor diagnosed asthma was reported by 27.6% of study participants. The mold sensitization rate was 10.4% and 14.9% among all participants and asthmatics, respectively. A list of mold specific positive reactions and the number of people testing positive to each mold is detailed in Table 2.

The frequency of mold/dampness by exposure category is described in Table 3. Water leaks, condensation, water damage, damp stains, or standing water was observed by 34.5% of the study population with a mean observation time of 9.1 months. A musty odor was reported by 27.4% (mean number of months 9.6) and observed mold or mildew reported by 29.8% (mean number of months observed 10.0) of the study population. Using an ordinal scale, 103 people (19.5%) answered yes to one of the exposure questions, 86 (16.3%) answered yes to two of the exposure questions, 80 (15.1%) answered yes to three of the exposure questions, 19 (3.6%) answered yes to four of the exposure questions, and 9 (1.7%) answered yes to all five exposure questions. In the workplace, 18.7% of respondents observed mold or mildew with a mean observation time of 14.1 months. Approximately, 12% of respondents reported regularly working in an occupation with mold (mean 15.2 months). After totaling the number of months exposed to each of the exposure questions, the mean number of months of exposure to any of the dampness indicators in either the home or workplace was 13.3.

Residential renovation activities are described in Table 4. 163 people (31.3%) reported living inside a home while it was being renovated and half (*n* = 262) took part in some type of renovation activity, although when asked what kind, the data were largely missing. Of those who took part in renovation activities, 119 people (48.6%) reported that they wore personal protective equipment at least 50% of the time; 32.7% reported no personal protective equipment use despite engaging in renovation work.

The relation between mold/dampness and mold reactivity was first assessed for each question individually. None of the exposures was individually related to the outcome (data not shown). Multivariable logistic regression models were run to assess the association between mold/dampness and mold positivity adjusting for age, gender, and asthma status (Table 5). In adjusted models, the relationship between mold/dampness exposure and cases of mold positive was nonsignificant whether exposure was measured on an ordinal scale (*P* = .64), or categorical scale (minimal exposure *P* = .09; moderate exposure *P* = .82; high exposure *P* = .83). When exposure was expressed as the total months of exposure to any of the five mold/dampness variables, the relationship was also nonsignificant (O.R = .99; 95%  C.I = .98–1.01; *P* = .46).

## 4. Discussion

When the federally built flood protection system failed, the New Orleans area was inundated by floodwaters. The majority of the population evacuated and returned to homes with varying degrees of flood damage. Over half of study respondents' homes had enough damage to require substantive repair. On average, respondents reported exposure to any of the dampness indicators for 13 months. The role of indoor dampness in triggering and exacerbating asthma and other respiratory symptoms has been documented in numerous studies; however, there is limited data on the relation of indoor dampness to mold sensitivity, particularly in populations living in areas with significant flooding events. The results of this analysis failed to find a significant relationship between any of the measures of mold/dampness exposure and sensitivity to mold allergens either in unadjusted or adjusted models. Furthermore, when asthma status was considered in the model, the results did not materially change. Thus, the finding of no association was robust across measures of exposure and across the study population. Although direct pre-post storm comparisons cannot be made, to explore the possibility that mold sensitivity was higher than expected in New Orleans residents, we compared our estimates to those obtained from general population estimates. To our surprise, we found the mold reactivity rate of 10% found in our sample equaled the rate in the general U.S. population [5]. Further, among asthmatics, the prevalence of mold reactivity, at 14.9%, was lower than rates found in both the general population and in other atopic populations [5, 24].

Exposure to molds and fungi can elicit a nonallergic response in persons not sensitized to mold/fungi allergen, with respiratory symptoms that are similar to those with an allergic response. Epidemiological studies in adults have shown comparable associations between home dampness and the occurrence of respiratory symptoms among allergic and nonallergic subjects, suggesting that damp-related symptoms in adults are not necessarily promoted by atopy [13]. Other studies, however, have shown a higher respiratory response and/or asthma diagnosis in those sensitized to mold allergen or with atopic heredity in relation to mold allergen [7]. Numerous reports of adverse health effects and reports of increased visits to health care providers due to allergic responses attributed to moldy environs have been reported in the post-Hurricane Katrina environment. It is possible that these persons experienced a nonallergic respiratory response to mold exposure which would not be captured by this design.

A limitation of the study is participant selection. Due to population fluctuation in the years immediately following Hurricane Katrina, retrospectively contacting patients was a challenge. The overall response rate was 52%. There was no way to determine whether the address recorded at the time of the clinic visit was relevant at the time the study was conducted. Therefore, the reason for nonresponse is unknown. While lower than optimal response rates may have introduced selection bias, analysis of responders versus nonresponders revealed no differences in sensitivity profile, age, gender, race, or geographic location. Another limitation is that minorities and those without health insurance are underrepresented, limiting the generalizability of study findings. Although over half the study subjects' homes incurred significant hurricane damage, survey data indicate the majority of respondents did not live for long periods of time in their homes during renovation. Due to the extent of damage incurred in most flooded homes, it seems reasonable that this pattern of habitation would reflect the general population experience. However, people at highest risk for mold sensitivity may be those with limited incomes who could not afford to stay out of their houses for long periods of time, who did significant amounts of renovation work, and who may not have access to health care. Studies targeting this subpopulation are needed. Finally, a potential limitation of this study is self-report of mold/dampness/musty exposure. However, our exposure assessment tools have been shown to be associated with objective measures in numerous studies and given that the findings were consistent for all three methods of exposure assessment utilized, the likelihood that information bias is responsible for our findings is minimized [5, 22, 23]. 

This study provides important information on the health of residents living in an area after widespread damage due to flooding. Over half the study population had significant home damage, 34% reported dampness/mold in their home, half reported engaging in renovation activities, and approximately one-third lived in a home undergoing renovation. Earlier research confirmed that potential exposure was high; both the indoor and outdoor environments were found to have extremely high fungal spore concentrations [15, 16, 25] prompting concern about development of mold sensitivity in the general population. Despite these concerns, the mold sensitivity rate was found to be typical of the population at large and was not found to be related to the amount of home damage or exposure to mold/dampness in the home. A strength of the study was the extensive exposure assessment which included duration of exposure in all households occupied over the course of the study as well as workplace exposure. Alternate explanations for the negative findings include lack of power due higher than anticipated exposure rates in both groups, 60% and 55.7% in disease and nondiseased, respectively, a nonatopic response or less than anticipated resident exposure because of the severity of flooding. This paradoxical situation of a protective effect of flooding may have occurred because homes were so badly damaged that they were uninhabitable until gutting mold contaminated material was complete.

## 5. Conclusion

We did not find elevated prevalence of mold sensitivity among those living in damp/moldy homes. Direct comparisons of mold sensitization rates pre- versus post-hurricane Katrina cannot be made in this study due to lack of pre-storm data, however, when comparing the overall rate of mold sensitivity in New Orleans to general population data and rates from similar sampling frames, we did not find an excess rate [5, 24]. These results along with results of earlier research [26] indicate no excess risk of adverse health effects in residents returning to live in New Orleans after the devastation of Hurricane Katrina.

## Figures and Tables

**Table 1:** Characteristics of the study population (*n* = 529).

Characteristic*	*n* (%)
Mean age in years (range)	41.5 (1–93)
Race	
White/Caucasian	414 (78.7)
African American	83 (15.8)
Hispanic/Latino	20 (3.8)
Asian	3 (0.6)
Other	6 (1.1)
Female Gender	355 (67.1)
Number of Residences since Katrina	
1	278 (52.5)
2	133 (25.1)
3	86 (16.2)
4 or more	32 (6.2)
Surface material or structural home repair needed	282 (53.3)
Doctor-diagnosed asthma	
Yes	141 (27.6)
No	352 (68.7)
Skin Prick Test Results	
Mold positive	55 (10.4)
At least one positive indoor allergen	194 (36.7)
Cat pelt positive	93 (17.6)
Mixed cockroach positive	24 (4.5)
Dog epithelium positive	22 (4.2)
Any dust mite positive	179 (34.1)
At least one positive outdoor allergen	221 (41.8)
Any Tree Pollen	146 (27.6)
Any Weed Pollen	119 (22.5)
Any Grass Pollen	141 (26.6)
At least one positive indoor or outdoor result	300 (56.7%)

*Variables not equaling 529 are due to missing data.

**Table 2:** Results of SPT for mold and the number of persons testing positive to each mold.

Type of Mold	Number Testing Positive
*Acremonium*	4
*Alternaria alternate*	14
*Aspergillus fumigates*	6
*Botrytis cinerea*	9
*Candida albicans*	0
*Chaetomium globosum*	0
*Cladosporium cladosporioides*	5
*Curvularia spp.*	20
*Epicoccum nigrum*	10
*Fusarium spp.*	5
*Gliocladium*	6
*Helminosporium*	15
*Mucor spp.*	2
*Neurospora spp.*	3
*Nigrospora spp.*	4
*Penicillium mixed*	9
*Phoma herbarum*	13
*Pullularia pullulans*	3
*Rhizopus spp.*	0
*Rhodotorula rubra*	0
*Smuts, Mixed*	0
*Stemphyllium spp.*	3
*Trichoderma*	2
*Trichophyton mentagrophytes*	1

**Table 3:** Dampness/mold in the home or workplace of study participants (*n* = 529).

Home Exposure Variables*	*n* (%)
*Q. 1 Water damage since Hurricane Katrina*	
Yes	180 (34.5)
No	324 (62.2)
Mean number of months exposed to water damage	9.1
*Q. 2 Musty odor since Katrina*	
Yes	143 (27.4)
No	354 (67.8)
Mean number of months exposed to musty odor	9.6
*Q. 3 Mold or mildew observed since Katrina*	
Yes	156 (29.8)
No	351 (67.0)
Mean number of months observing mold or mildew	10.0

Workplace Variables*	

*Q. 4 Mold or mildew observed since Hurricane Katrina*	
Yes	98 (18.7)
No	369 (70.6)
Mean number of months mold/mildew observed	14.1
*Q. 5 Ever worked in an occupation regularly exposed to mold*	
Yes	59 (11.9)
No	367 (74.0)
Mean number of months working in occupation with mold	15.2

Dampness index	

(1) answered yes to 1 question	103 (19.5)
(2) answered yes to 2 questions	86 (16.3)
(3) answered yes to 3 questions	80 (15.1)
(4) answered yes to 4 questions	19 (3.6)
(5) answered yes to all 5 questions	9 (1.7)
Mean Months exposed to any of the dampness indicators	13.3

*totals not equaling 529 are due to missing data.

**Table 4:** Residential renovation activities.

Renovation activity	*n* (%)
House underwent renovation while person lived in it	
Yes	163 (31.3)
No	355 (68.1)
Participated in renovation activities	262 (49.9)
Mean months of renovation participation	3.9
Protective equipment worn while renovating*	
100% of the time	28 (11.4)
75% of the time	37 (15.1)
50% of the time	37 (15.1)
25% of the time	46 (18.8)
0% of the time	80 (32.7)
Not applicable	17 (6.9)

*of those participating in renovation activities.

**Table 5:** Multivariable analysis of mold/dampness indicators and allergic sensitization to mold adjusted for age and gender.

Mold/Dampness Indicator	Sensitization to Mold								
	O.R.	95% C.I.	*P*-value	O.R.	95% C.I.	*P*-value	O.R.	95% C.I.	*P*-value
Mold Exposure (0–5)	.95	75–1.19	.64						
Minimal, Moderate, Highly exposed*									
Minimal exposure				1.85	.90–3.83	.09			
Moderate exposure				1.09	.53–2.25	.82			
High exposure				.85	.18–3.92	.83			
Total Months of Exposure							.99	.98–1.01	.52
Doctor-diagnosed Asthma	1.0	1.0–1.0	.53	1.00	1.0–1.0	.46	1.0	1.0–1.0	.53

*no reported exposure is the comparison group.
